# Fluorescence Microscopy Assay to Measure HIV-1 Capsid Uncoating Kinetics *in vitro*

**DOI:** 10.21769/BioProtoc.3297

**Published:** 2019-07-05

**Authors:** Chantal L. Márquez, Derrick Lau, James Walsh, K. M. Rifat Faysal, Michael W. Parker, Stuart G. Turville, Till Böcking

**Affiliations:** 1 EMBL Australia Node in Single Molecule Science and ARC Centre of Excellence in Advanced Molecular Imaging, School of Medical Sciences, UNSW, Sydney, Australia; 2 St. Vincent’s Institute of Medical Research, Australia; Bio21 Molecular Science and Biotechnology Institute, University of Melbourne, Australia; 3 The Kirby Institute, UNSW, Sydney, Australia

**Keywords:** Retrovirus, HIV, Capsid stability assay, Uncoating, CypA, Viral particles, CA

## Abstract

The stability of the HIV-1 capsid and the spatiotemporal control of its disassembly, a process called uncoating, need to be finely tuned for infection to proceed. Biochemical methods for measuring capsid lattice disassembly in bulk are unable to resolve intermediates in the uncoating reaction. We have developed a single-particle fluorescence microscopy method to follow the real-time uncoating kinetics of authentic HIV capsids *in vitro*. The assay utilizes immobilized viral particles that are permeabilized with the a pore-former protein, and is designed to (1) detect the first defect of the capsid by the release of a solution phase marker (GFP) and (2) visualize the disassembly of the capsid over time by “painting” the capsid lattice with labeled cyclophilin A (CypA), a protein that binds weakly to the outside of the capsid. This novel assay allows the study of dynamic interactions of molecules with hundreds of individual capsids as well as to determine their effect on viral capsid stability, which provides a powerful tool for dissecting uncoating mechanisms and for the development of capsid-binding drugs.

## Background

The human immunodeficiency virus-1 (HIV-1) is a lentivirus that replicates in CD4-positive immune cells. HIV-1 copies its genomic RNA into DNA, which integrates into the host genome to establish a persistent infection within the host (Lusic and Siliciano, 2017). In the mature virion, the viral genome is enclosed within the capsid, a conical shell formed by the viral capsid protein (CA) organized into ~250 hexamers and exactly 12 pentamers (Briggs, *et al.*, 2003 and 2004). After fusion of the viral envelope with the host cell membrane, the capsid is released into the cytoplasm where it fulfills multiple essential functions, making capsid an attractive drug target. It not only protects the viral genome from degradation, but is also involved in influencing many viral post fusion events, including cytoplasmic transport, reverse transcription, nuclear import and integration site targeting (Ocwieja *et al.*, 2011; Schaller *et al.*, 2011; Lahaye *et al.*, 2013; Rasaiyaah *et al.*, 2013; Matreyek *et al.*, 2013; Jacques *et al.*, 2016; Dharan *et al.*, 2016; Sowd *et al.*, 2016; Burdick *et al.*, 2017). The viral capsid must ultimately disassemble in a gradual process termed uncoating to release the viral DNA. To regulate capsid stability and uncoating, HIV-1 capsid binds different sets of capsid-binding host molecules in the cytoplasm, at the nuclear pore complex and inside the nucleus. For example, the peptidylprolyl isomerase cyclophilin A (CypA) binds to multiple CypA bindings loops that are exposed on the outside of the capsid, which may stabilize the capsid during transport from the cell periphery to the nucleus. A roadblock to dissecting the order of events and precise effects of host factors and capsid-binding drugs on uncoating is the unavailability of methods to measure the kinetics of the uncoating reaction at the single-particle level. Most of the current biochemical methods to study HIV capsid stability, such as *in vitro* uncoating of isolated cores (Kotov *et al.*, 1999; Forshey *et al.*, 2002; Shah and Aiken, 2011), the fate of capsid assay (Stremlau *et al.*, 2006; Yang *et al.*, 2014), or cyclosporin A (CsA) washout assay (Perez-Caballero *et al.*, 2005; Hulme *et al.*, 2011; Hulme and Hope, 2014) rely on observing the average behavior of large numbers of viral cores, which obscures the identification of intermediates in the uncoating process. Fluorescence microscopy methods can resolve the uncoating of individual capsids in the cytoplasm (Campbell *et al.*, 2008; Pereira *et al.*, 2011; Ma *et al.*, 2016; Francis *et al.*, 2016; Mamede *et al.*, 2017; Francis and Melikyan, 2018), but complementary *in vitro* methods that allow higher throughput measurements and detailed kinetic studies under defined conditions are still missing.

We recently developed a novel fluorescence imaging method to follow the real-time uncoating kinetics of individual HIV capsids *in vitro* and applied this method to reveal how capsid disassembly is modulated by different host factors and small molecules (Mallery *et al.*, 2018; Marquez *et al.*, 2018). In our method, HIV-1 virus-like particles loaded with GFP as a content marker are immobilized on a coverslip for observation by total internal reflection (TIRF) microscopy. Upon permeabilization, the pool of GFP retained by the viral membrane is released while the pool of GFP trapped inside the capsid remains. Simultaneously, fluorescence-labeled CypA contained in the bulk solution can access and “paint” the capsid by binding to sites on its exterior surface. CypA binding is reversible, whereby the intensity of the CypA paint signal (*i.e.*, the number of CypA molecules bound at equilibrium) is proportional to the size of the CA lattice. At the concentrations used in this assay (up to 1 μM, *i.e.*, about 10-fold lower than cytosolic concentrations), CypA binding has no measurable effect on capsid stability. The assay allows the measurement of the two processes involved in capsid uncoating for each particle in the field of view: (1) The sudden loss of the residual GFP signal pinpoints the precise time at which the first defect appears in the capsid, and (2) the gradual decay of the CypA paint signal reveals the kinetics of CA lattice disassembly thereafter. The method has several advantages: it studies authentic HIV capsids *in vitro*, utilizing small sample volumes in the low μl range; hundreds of individual capsids are monitored in a single experiment and analyzed as single particles, allowing the identification of intermediates in the disassembly pathway that are averaged out in ensemble experiments; it allows screening of the effect of several molecules and proteins on capsid stability as well as the study of their binding kinetics. Our system has potential as a medium-throughput assay for screening compounds that target the stability of the HIV-1 capsid lattice. Furthermore, the approach can readily be applied to study capsid uncoating for other enveloped viruses.

## Materials and Reagents

- Viral particle preparation
  1. T25 cell culture flask
  2. Microwell plate
  3. 10 cm^2^ culture dishes (BD Biosciences, catalog number: 353803)
  4. Poly-L-lysine-coated glass-bottom (175 μm thickness) 96-well plates (Greiner Sensoplate, Sigma, catalog number: M4187)
  5. HEK-293T cells (ATCC, catalog number: CRL-3216)
  6. Dulbecco’s Modified Eagle Medium (DMEM) (Life Technologies, Invitrogen, catalog number: 11965-092)
  7. Fetal bovine serum (FBS) (Sigma-Aldrich, catalog number: F2442-500ML)
  8. 1x PBS (Gibco, catalog number: 10010031)
  9. 1x trypsin-EDTA (Gibco, catalog number: 15400054)
  10. Plasmid, psPAX2 (NIH AIDS Reagent Program, catalog number: 11348)
  11. Plasmid, pNL4.3-iGFP-Env (Hubner *et al.*, 2007; Aggarwal *et al.*, 2012)
  12. Polyethylenimine (PEI Max) reagent (Polysciences, catalog number: 9002-98-6)
  13. 0.9% w/v sodium chloride (Sigma-Aldrich, catalog number: S8776)
  14. EZ-Link Sulfo-NHS-LC-LC-Biotin (Thermo Scientific, catalog number: 21338)
  15. HEPES (Sigma-Aldrich, catalog number: H3375-250G)
  16. NaCl (Chem Supply, catalog number: SA046-5KG)
  17. Cell culture media (see Recipes)
  18. HBS pH 7.5 and 7.0 (see Recipes)
- AF568-CypA preparation
  1. 0.22 μm syringe filter
  2. Amicon-15 Ultra centrifugal filtration device (10k MWCO, Merck, UFC901024)
  3. BL21 (DE3) *E. coli* cells
  4. Plasmid, pETMCSI-hCypA (pBH964) (Ozawa *et al.*, 2004)
  5. Pierce Coomassie Plus (Bradford) Assay Kit (Thermo Scientific, catalog number: 23236)
  6. Alexa-Fluor 568-C5-maleimide dye (Thermo Fisher Scientific, catalog number: A20341)
  7. AF568-CypA (see Procedure)
  8. Complete EDTA-free EASYpack, Protease Inhibitor Cocktail tablets (Roche, catalog number: 04693132001)
  9. LB medium (MP Bio, catalog number: 113002041)
  10. Isopropyl β-D-thiogalactopyranoside (IPTG)
  11. Liquid nitrogen
  12. Ampicillin powder (Thermo Scientific, catalog number: BP1760-25)
  13. 1% v/v acetic acid diluted from glacial stock (Ajax Finechem, catalog number: AJA1-2.5LPL)
  14. BSA (Bovogen Biologicals, catalog number: BSAS-NZ 0.1)
  15. DTT (Sigma-Aldrich, catalog number: 43815-25G)
  16. TCEP (Sigma-Aldrich, catalog number: C4706-2G)
  17. HEPES (Sigma-Aldrich, catalog number: H3375-250G)
  18. NaN_3 _(Sigma-Aldrich, catalog number: S2002-500G)
  19. NaCl (Chem Supply, catalog number: SA046-5KG)
  20. Lysozyme (Sigma-Aldrich, catalog number: 62971-10G-F)
  21. MOPS (Sigma-Aldrich, catalog number: M1254-1KG)
  22. Tris (Ajax Finechem, catalog number: AJA2311-5KG)
  23. Glycerol (Ajax Finechem, catalog number: AJA242-2.5LGL)
  24. Lysis buffer (see Recipes)
  25. AIEX buffer A (see Recipes)
  26. AIEX buffer B (see Recipes)
  27. CIEX buffer A (see Recipes)
  28. CIEX buffer B (see Recipes)
  29. CypA storage buffer (see Recipes)
  30. AF568-CypA storage buffer (see Recipes)
- Microfluidic device preparation
  1. Plastic weigh-boat (Sigma, or equivalent)
  2. Tubing Intramedic 0.043 in. (Becton Dickinson, catalog number: 427406)
  3. Disposable needles, 1 mm OD (Becton Dickinson, or equivalent)
  4. Blu tack (Bostik, or equivalent)
  5. Round glass coverslips, 25 mm diameter (Paul Marienfeld GmbH & Co KG, catalog number: 0117650)
  6. Biotinylated poly-L-lysine-g-poly-ethylene glycol (SuSoS AG, PLL-g-PEG-biotin, PLL(20)-g[3.5]-PEG(2)/PEG(3.4)-biotin(20%))
  7. Streptavidin (Life Technologies Australia, catalog number: 434301)
  8. Sylgard184 silicone elastomer kit (Dow Corning)
  9. Absolute ethanol (Ajax Finechem, catalog number: AJA214-2.5LGL)
  10. 1 M NaOH (Ajax Finechem, catalog number: AJA482-500G)
  11. Isopropyl alcohol (Thermo Scientific, catalog number: AJA214-2.5LGL)
  12. Tris (Thermo Scientific, catalog number: AJA2311-5KG)
  13. EDTA (Ajax Finechem, catalog number: AJA180-500G)
  14. NaN_3 _(Sigma-Aldrich, catalog number: S2002-500G)
  15. Tween 20 (Sigma-Aldrich, catalog number: P5927-500ML)
  16. BSA (Bovogen Biologicals, catalog number: BSAS-NZ 0.1)
  17. Blocking buffer (see Recipes)
- Imaging assay
  1. Bacterial pore-forming protein for viral membrane permeabilization
**Option 1:** Recombinant cysteine-less (C459A) perfringolysin O (PFO) with N-terminal His-tag was supplied by Michael Parker.
The protocols for expression and purification of recombinant PFO C459A have been described previously (Shepard *et al.*, 1998; Wade *et al.*, 2015). Production and handling of PFO should be done in accordance with local occupational health and safety regulations for handling toxins. Store frozen in small aliquots at -80 °C. After thawing an aliquot, store at room temperature for up to two months. The protein loses activity when stored at 4 °C or during repeated freeze-thaw cycles.
**Option 2:** Alternatively, streptolysin O (SLO) (Sigma-Aldrich, catalog number: S5265) can be used for permeabilization of viral particles (unpublished data).
    Handling of SLO should be done in accordance with local occupational health and safety regulations for handling toxins. Dissolve the protein in PBS containing 2 mM TCEP to give a concentration of ~8 μM, prepare small aliquots, freeze in liquid nitrogen and store frozen at -80 °C. Thaw a fresh aliquot on the day of use. Unlike PFO C459A, wild type SLO loses activity in solution as a result of oxidation (disulfide formation).
  2. Protocatechuic acid (Sigma-Aldrich, catalog number: 37580-25G-F)
  3. Protocatechuate-3,4-dioxygenase (Sigma-Aldrich, catalog number: P8279-25UN)
  4. Trolox (Sigma-Aldrich, catalog number: 238813-1G)
- General
  1. Sharp blades (TechnoCut, or equivalent)
  2. Serological pipettes, 5,10 and 25 ml (Corning, or equivalent)
  3. Microcentrifuge tubes (Eppendorf, or equivalent)
  4. Sterile 15- and 50-ml conical tubes (Falcon, or equivalent)
  5. Sterile pipette tips (Eppendorf, or equivalent)
  6. Disposable syringes, 1 and 3 ml (Becton Dickinson, or equivalent)
  7. Milli-Q Water (from Millipore Milli-Q Integral 5 Water Purification System, or equivalent)

## Equipment

- Viral particle preparation
  1. 10-ml super loop
  2. Tissue culture incubator, humidity, temperature and CO_2_ regulated (Thermo Fisher Scientific, model: 3110, or equivalent)
  3. Biosafety cabinet (Thermo Fisher Scientific, model: 1323TS, or equivalent)
  4. HiPrep 16/60 Sephacryl S-500 HR column (GE Healthcare, catalog number: 28-9356-06)
  5. Fast protein liquid chromatography (FPLC) system including injector, one pump, UV-detector, fraction collector (GE Healthcare, ÄKTA pure, or equivalent)
  6. 4 °C refrigerator
  -20 °C freezer
  -80 °C freezer
- AF568-CypA preparation
  1. 5 ml HiTrap Q HP column (GE Healthcare Life Science, catalog number: 17115401)
  2. 5 ml HiTrap SP HP column (GE Healthcare Life Science, catalog number: 17115201)
  3. Zeba desalting spin column (Thermo Fisher Scientific, catalog number: 89883)
  4. CelluSep T1 regenerated tubular membrane MWCO 3,500 Da (CelluSep, catalog number: 501546)
  5. Branson Sonifier 250 Probe Sonicator mounted with a tapered microtip
- Microfluidic set-up and capsid uncoating imaging
  1. TIRF Microscope, see specifications in Part IV
  2. Magnetic chamber for 25 mm round coverslips (Chamlide chamber, model: CM-B25-1)
  3. Syringe pump (New Era Pump System Inc., model: NE-1002X)
  4. Mold (*e.g.*, prepared by photolithography on a silicon wafer or machined from aluminum) for making microfluidic devices; each device contains 5 flow cell channels; channel dimension 1,100 μm x 800 μm x 40 μm (length x width x height)
  5. Plasma cleaner (Harrick Plasma, catalog number: PDC-32G)
  6. Ceramic coverslip holder
  7. Vacuum desiccator
  8. Harris Uni-Core 1.0 mm punch
  9. Oven
  10. Andor iXon 888 EMCCD cameras
- General
  1. Pipettes (Gilson, catalog number: F167700, or equivalent)
  2. Refrigerated tabletop centrifuge (Thermo Fisher Scientific, model: 75004524, or equivalent)
  3. Tabletop centrifuge (Eppendorf Centrifuge, model: 5417R, or equivalent)
  4. NanoDrop 1000 spectrophotometer or equivalent machine that can read UV-vis spectra
  5. Rotator (Benchmark Scientific, model: R5010, or equivalent)

## Software

*Note: The following software package can be used individually or in combination for image analysis.*

1. FIJI image analysis software (Schindelin *et al.*, 2012; https://imagej.net/Fiji/Downloads)
2. Matlab (Mathworks, https://www.mathworks.com/products/matlab.html )
3. JIM Immobilized Microscopy analysis package ( https://github.com/lilbutsa/JIM-Immobilized-Microscopy-Suite )

## Procedure

**Part I: HIV-1 viral particle preparation**

- Produce HIV-1 viral particles (2.5 days)
*Note: The combination of plasmids used in this protocol results in the production of virus-like particles that lack envelope proteins and are non-infectious; procedures should be done in accordance with local biosafety regulations for producing virus-like particles.*
  1. In a 15 ml conical tube, prepare DNA solution for transfection by mixing 6.6 μg of pNL4.3-iGFP-Env plasmid, 3.3 μg of psPAX2 plasmid, 60 μl of 1 mg/ml PEI max solution in a final volume of 500 μl of 0.9% (w/v) sodium chloride. Incubate the mixture for 30 min at room temperature to allow formation of DNA:PEI complexes.
  2. Split the HEK-293T cell culture as follows:
    - Remove the culture medium and wash the cell monolayer with 1x PBS. Add 1x trypsin-EDTA (typically 1 ml for a T25 cell culture flask) and incubate for 5 min at 37 °C.
    - Stop the trypsin digestion by adding new culture media and transfer the cell suspension to a 15 ml conical tube.
    - Take a sample to count cells and determine the total number of cells in the tube. Centrifuge the cell suspension at 300 *× g* for 5 min at room temperature to pellet the cells and discard the supernatant. Resuspend the cells in the appropriate volume of fresh culture media to obtain a concentration of 7 x 10^6^ cells/ml.
  3. Gently add 1 ml of cell suspension to the DNA:PEI mix and incubate for 5 min at room temperature.
  4. Plate the cells:DNA:PEI mixture drop by drop in a 10 cm^2^ culture dish containing 6.5 ml of culture media. Slightly shake the dish to distribute the cells homogeneously. Incubate at 37 °C with 5% CO_2_ for 48 h.
  5. Collect the virus-containing supernatant in a 15 ml conical tube and centrifuge at 2,100 *× g* for 10 min at 4 °C to remove cellular debris.
  6. Collect the supernatant and transfer it to a new conical tube. The final volume of the cleared virus-containing medium should be around 7 ml.
*Note: To verify the presence of fluorescent HIV particles in the supernatant, dilute 1 in 100 in PBS and then spinoculate 200 μl of the virus preparation onto a poly-L-lysine-coated glass-bottom 96-well plate well at 1,200 × g for 60 min at 4 °C, and inspect for fluorescent dots with a 60x objective in a fluorescence microscope. The number of viral particles per surface area can be determined by counting the number of fluorescent dots in at least 4 fields of view. The particle concentration can then be obtained by multiplying the number of particles per surface area with the surface area of the well (to obtain the number of particles per well) and then dividing this number by the volume of supernatant added to the well (0.2 ml).*
- Biotinylate the HIV-1 viral particles (2 h)
  1. Dissolve ~1 mg of EZ-Link^TM^ Sulfo-NHS-LC-LC-Biotin in 1 ml of HBS pH 7.5 in a microcentrifuge tube.
  2. Add the biotinylation reagent to the virus-containing medium and incubate in the dark for 90 min at 4 °C with gentle rotation.
- Purify the biotinylated HIV-1 viral particles by size exclusion chromatography (1 day)
  1. Connect a HiPrep 16/60 Sephacryl S-500 HR size exclusion chromatography column to the FPLC system. All solutions used for FPLC should be passed through a filtration membrane with 0.2 μm pore size for removal of particulates and to degas the solutions.
  2. Replace the storage solution (usually 20% ethanol) with 2 column volumes (CV) of purified water and then equilibrate the column with 2 column volumes (CV) of HBS pH 7.5. Monitor the absorption at 280 nm. Flow rate: 1 ml/min.
*Note: This step should be started the day before collecting the viral particles as it takes ~8 h for the column to be equilibrated.*
  3. Load the biotinylated viral particles (~6.5 ml) onto the column using a 10-ml super loop. Monitor the absorption at 280 nm. Flow rate: 0.5 ml/min.
  4. Elute the sample with HBS pH 7.5 and collect 1 ml fractions until a total of 1.5 CV is reached (~6 h). A representative elution profile is shown in Figure 1.
  5. Combine the fractions corresponding to the first small peak that contain the viral particles (typically around fractions 34-39 or C10-D3 on a microwell plate).
  6. Purified biotinylated viral particles can be used within 7 days if they are stored at 4 °C, or you can make 200-500 μl aliquots and store at -80 °C (no flash freezing in liquid nitrogen needed). Frozen samples can be thawed on ice for use.
  7. Wash the column with 2 CV of purified water and then with storage solution.
    **Figure 1.**
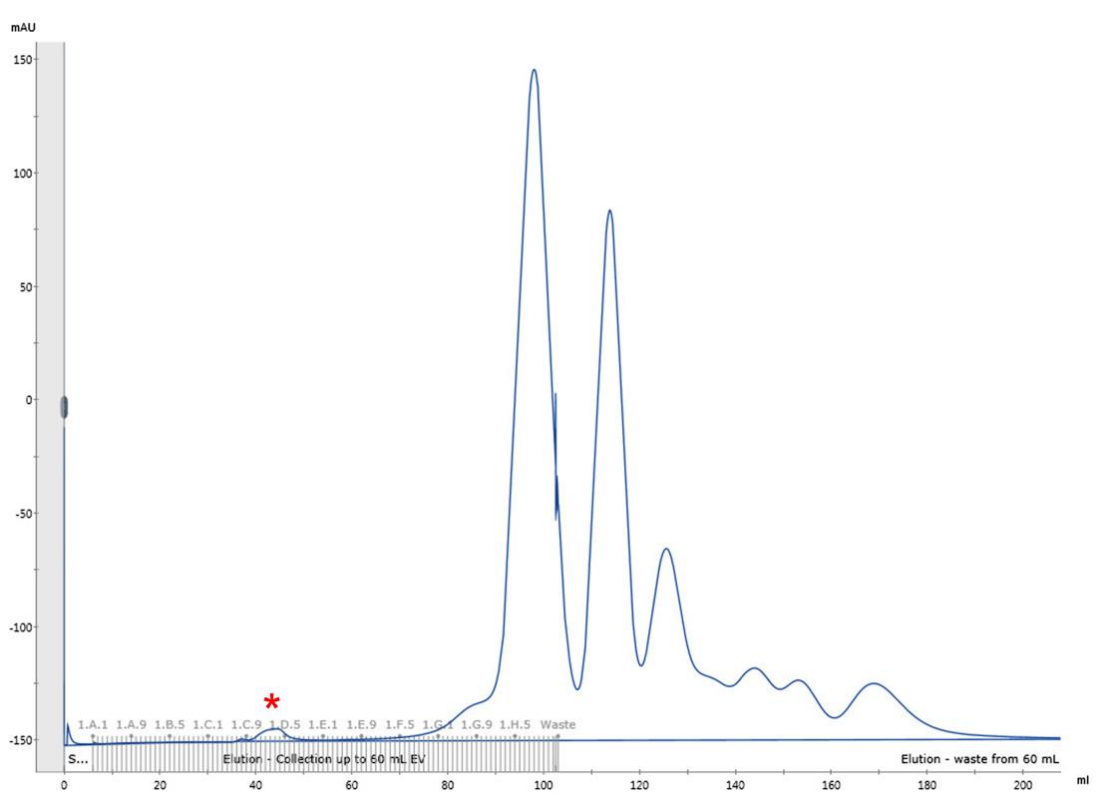
Representative elution profile of biotinylated HIV-1 viral particles by size exclusion chromatography. Chromatographic separation of HIV-1 viral particles from culture media proteins. The first small peak (fractions C10-D3, marked with a red asterisk) corresponds to the HIV-1 viral particles.
**Part II: Preparation AF568-CypA for lattice painting**

- Express and purify human CypA (3-4 days)
  1. Grow BL21 (DE3) *E. coli* cells expressing human CypA in a pET expression vector in LB medium supplemented with ampicillin (final 100 μg/ml) at 37 °C, 180 rpm, overnight as a starter culture.
  2. Inoculate 500 ml of fresh LB medium supplemented with ampicillin (final 100 μg/ml) with 5 ml of starter culture.
  3. Monitor the absorbance at 600 nm (OD_600_). Induce protein expression by addition of isopropyl β-D-thiogalactopyranoside (IPTG) to a final concentration of 1 mM when the OD_600_ reaches a value of 0.6. Allow protein expression to proceed for 3 h at 37 °C, 180 rpm.
  4. Pellet the bacterial cells by centrifugation (6,000 *× g*, 20 min at 4 °C) and discard the supernatant.
  5. Resuspend the cell pellet in a cold lysis buffer (typically use 15 ml of buffer per 500 ml of culture pellet) and lyse cells by sonication on ice (50% duty cycle, setting 3, 15 s on/off per cycle, 12 cycles).
*Note: Cells can be lysed with any other sonicator or method for lysis of bacterial cells.*
  6. Centrifuge the lysate at 43,000 *× g* for 30 min at 4 °C, recover the supernatant and filter it through a syringe filter (0.22 μm).
  7. Purify the sample by subtractive anion exchange chromatography using 2 x 5 ml HiTrap Q HP columns connected in tandem. First, wash the columns with 5 CVs of AIEX buffer A, followed by 5 CVs of AIEX buffer B and finally equilibrate with 5 CVs of AIEX buffer A at 5 ml/min. Load the sample and collect the flowthrough in 1 ml fractions. Keep eluting with extra ~5 ml of AIEX buffer A to recover the entire flowthrough. Flow rate: 1 ml/min.
  8. Check each flowthrough fraction on reducing SDS-PAGE and combine the fractions that contain CypA. Adjust pH to 5.8 with 1% v/v acetic acid using a pH probe to measure the pH. A representative elution profile is shown in Figures 2A and 2C.
  9. Remove aggregates by centrifugation (43,000 *× g*, 1 h, 4 °C) and collect the supernatant.
  10. Purify CypA contained in the supernatant further using cation exchange chromatography using a 5 ml HiTrap SP HP column washed with 5 CVs of CIEX buffer A, followed by 5 CVs of CIEX buffer B, and finally equilibrated with 5 CVs of CIEX buffer A at 5 ml/min. Inject the sample onto the equilibrated column at 1 ml/min, then wash with 2 CVs of CIEX buffer A. Elute bound CypA with a 0-1 M linear gradient of NaCl (0%-100% CIEX buffer B) over 20 CVs at 5 ml/min. The CypA peak will elute at 20-25% CIEX buffer B. A representative elution profile is shown in Figures 2B and 2D.
  11. Dialyze the fractions containing purified CypA against CypA storage buffer for 3 x 4 h using dialysis tubing (*e.g.*, CelluSep T1 regenerated membrane) with a molecular weight cut-off (MWCO) of 3,500 Da.
  12. Determine the concentration of protein using the Bradford assay with BSA as a standard.
  13. If necessary, concentrate the protein using an Amicon-15 Ultra centrifugal filtration device (10k MWCO). The final recommended concentration should be 100-300 μM (1.8-5.4 mg/ml).
  14. Use immediately or aliquot and flash freeze in liquid nitrogen for storage at -80 °C.
    **Figure 2.**
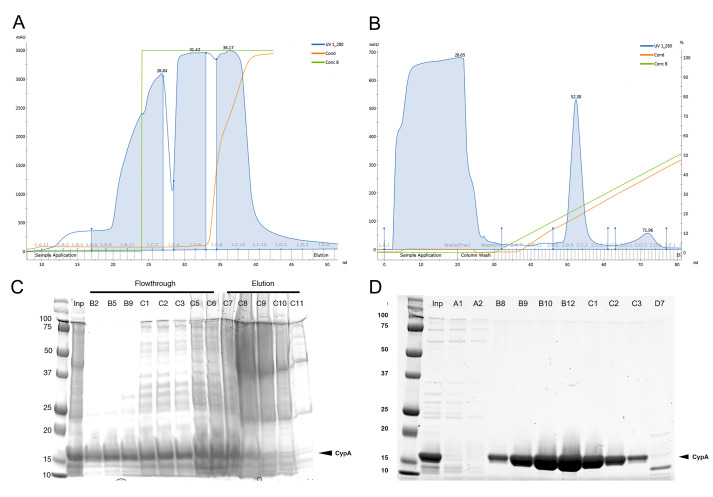
Purification of CypA by ion exchange chromatography. Elution profiles and corresponding SDS-PAGE analysis with Coomassie Blue staining of selected fractions from anion (A, C) and cation (B, D) exchange chromatography steps for CypA purification.
- Fluorescence labeling of CypA (4 h)
  1. Exchange the buffer of purified recombinant CypA using a Zeba desalting spin column equilibrated with PBS containing 0.1 mM TCEP to remove the DTT contained in the CypA storage buffer.
  2. Mix purified CypA with a 4-fold molar excess of Alexa Fluor 568-C5-maleimide dye in PBS pH 7.4 and incubate for 10 min at room temperature. A typical reaction for labeling of 130 μl of 100 μM recombinant CypA requires 5 μl of 10 mM Alexa Fluor 568-C5-maleimide.
  3. Add DTT solution (1 M) to a final concentration of 10 mM to quench unconjugated dye.
  4. Remove the free dye by exchanging the buffer using a Zeba desalting spin column equilibrated with AF568-CypA storage buffer.
  5. Measure a UV-visible absorption spectrum of AF568-CypA using a NanoDrop 1000 or equivalent instrument to determine the absorption at 280 nm (*A*_280_) and the absorption at wavelength of maximum dye absorbance (*A*_dye_, ~578 nm for Alexa Fluor 568). Calculate the protein concentration (*c*_CypA_) using the following formula: *c*_CypA_ = (*A*_280_ - CF x *A*_dye_)/*ε*_CypA_, where CF is a dye-specific correction factor (CF = 0.45 for Alexa Fluor 568) and *ε*_CypA_ is the molar extinction coefficient of CypA at 280 nm (*ε*_CypA_ = 8730 M^-1^cm^-1^). Calculate the degree of labeling (DOL) using the following formula: DOL = (*A*_dye_/*ε*_dye_)/*c*_CypA_, where *ε*_dye _is the molar extinction coefficient of the dye at its absorbance maximum (*ε*_dye_ = 91300 M^-1^cm^-1^ for Alexa Fluor 568). AF568-CypA prepared by this protocol should have a degree of labeling close to 1 fluorophore per protein.
  6. Prepare small aliquots, flash freeze in liquid nitrogen and store at -40 °C. Typically CypA-AF568 are stored in 2-4 μl at ~100 μM.
**Part III: Microfluidic flow cells set-up**

- Prepare polydimethylsiloxane (PDMS) microfluidic devices (1.5 days)
  1. Prepare 30 ml of the transparent polymer mixture (elastomer/curing agent ratio 10:1 w/v) in a plastic weigh-boat.
  2. Mix thoroughly (avoid formation of bubbles) and then pour over the silicon microlithography wafer mold (Figure 3A).
  3. Place the silicon mold containing the PDMS mix in a vacuum desiccator and degas under reduced pressure for 20 min to remove all air bubbles before curing.
  4. Cure the PDMS overnight at 70 °C in an oven. The cured PDMS should be firm to the touch.
  5. Carefully remove the PDMS devices from the mold by using a sharp blade (scalpel). Make sure you cut them into square pieces with dimensions that fit into the magnetic chamber used for imaging (see below).
  6. Place the PDMS block onto a clean surface with the channels facing up and punch holes for inlets and outlets at opposite ends of each channel by pushing a Harris Uni-Core 1.0 mm punch through the PDMS device. Remove polymer plugs from the holes.
  7. Clean the PDMS devices by immersion in water, isopropyl alcohol (100%) and water (10 min each step).
  8. Dry PDMS devices with lint-free tissue and store in a clean container with the channels facing up.
- Clean glass coverslips (3 h)
  1. Place round glass coverslips in a coverslip holder and wash them by sonication in absolute ethanol for 30 min followed by sonication in 1 M NaOH for 30 min.
  2. Rinse the coverslips by softly shaking the cover glass holder in a large beaker filled with ultrapure water. Repeat three times with fresh water each time. Handle carefully as the coverslips can stick together, fall out of the holder or break.
  3. Wick away the excess of water from the coverslips in the holder with a lint-free tissue without touching the coverslip surfaces. Then place the holder with coverslips into a heated oven for at least 30 min for drying.
  4. Use cleaned coverslips immediately or store them in a vacuum desiccator for use within a month.
- Assemble the microfluidic flow cells (1-2 h)
  Depending on the number of experiments you are planning to do, assemble one or more PDMS devices. We recommend always preparing one extra device in case the glass coverslip breaks, the channels leak or something else happens.
  1. Clean the channels of a PDMS device with isopropanol using a lint-free tissue. Let the surface dry.
  2. Place a coverslip (in a ceramic holder) and a PDMS inside a plasma cleaner and treat them with an air plasma for 3 min. The channels should be facing up.
  3. Carefully mount the PDMS device on the center of the coverslip with the channels facing the glass. Apply gentle, uniform pressure with your hand to ensure a uniform seal but without breaking the glass slide. To keep the coverslip clean, always place it on top of a lint-free tissue. The assembled device is shown in Figure 3B.
  4. Place the assembled microfluidic device in an oven at 70 °C for 15 min to improve bonding between the glass and the PDMS.
  5. Use immediately or store the devices in a clean container for use within 2 weeks.
    **Figure 3.**
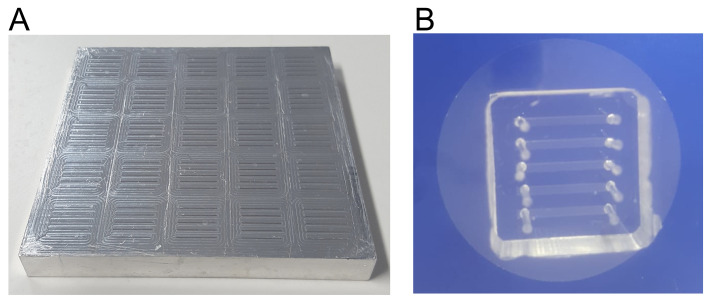
Microfluidic devices. A. Photo of the silicon microlithography wafer mold. B. Microfluidic device consisting of a PDMS block (with channels and holes for tubing) adhered to a glass coverslip.
- Prepare inlet and outlet tubing (0.5 h)
  1. For inlets, cut ~5-7 cm long pieces of tubing. Do not reuse inlet tubing.
  2. For outlets, cut ~10-13 cm long pieces of tubing. Connect a blunted needle (1 mm OD) to one of the ends. To reuse them, rinse the tubing with distilled water using a syringe. Dry by drawing air through the tubing.
**Part IV: Imaging assay**

- Microscope set-up
Images are acquired on a TIRF microscope. Our home-built system (designed by Philip Nicovich) is based on an ASI-RAMM frame (Applied Scientific Instrumentation) with a Nikon 100x CFI Apochromat TIRF (1.49 NA) oil immersion objective. Lasers were incorporated using the NicoLase system (Nicovich *et al.*, 2017). Images were captured on two Andor iXon 888 EMCCD cameras (Andor Technology Ltd) and 300 mm tube lenses were used to give a field of view of 88.68 μm x 88.68 μm at Nyquist sampling frequency (86 nm per pixel). The TIRF angle was adjusted to obtain ~200 nm penetration depth.
*
Note: The relationship between TIRF angle and penetration depth and methods to empirically determine penetration depth are discussed in the following reference (Fish, 2009).
*
- Capture viral particles onto the flow cell surface
  1. With a P20 micropipette, gently inject a solution of biotinylated poly-L-lysine-g-poly-ethylene glycol (1 mg/ml) into one of the ports of each flow channel until the solution reaches the other port (~3 μl). Incubate for 30 min at room temperature.
  2. Mount an assembled microfluidic device in a magnetic chamber and connect inlet and outlet tubing pieces to the opposite ports of each flow channel.
  3. Place the inlet tubing of all channels into a microcentrifuge tube filled with ultrapure water (Figure 4A). Use removable adhesive to position the tube close to the device.
    **Figure 4.**
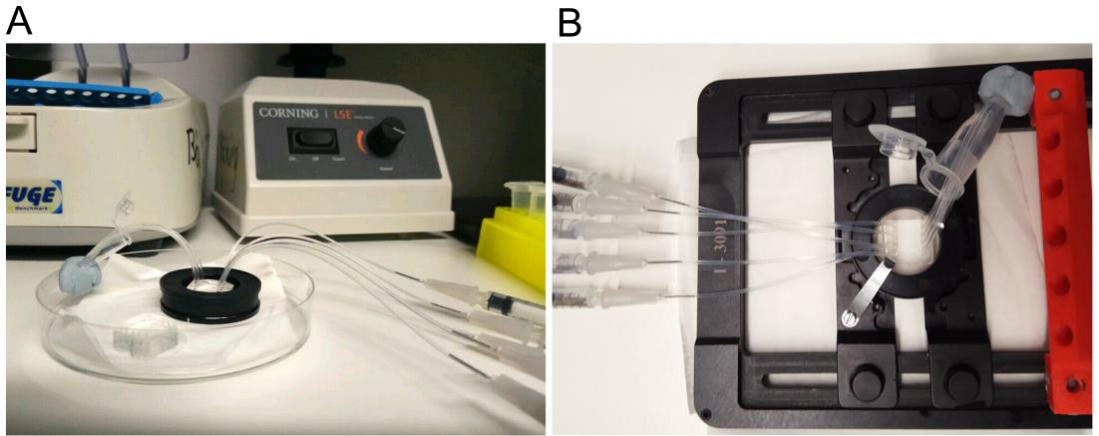
Setup for flow cell surface preparation. Picture of an assembled microfluidic device mounted on the magnetic holder in a (A) Petri dish or (B) on the microscope stage. Inlets (short tubing) are dipped into a microcentrifuge tube with HBS pH 7.5 while outlets (long tubing) are connected to a syringe.
  4. Attach a 1 ml syringe to an outlet tubing and carefully rinse the channel by drawing distilled water through the tubing (~200 μl each). Repeat for all channels. You can use the same syringe for all outlets (switching from one to the next) or connect a separate syringe to each outlet for ease of use.
  5. Remove the inlets from the water and remove the water from the channels by drawing air through them.
  6. Draw a solution of 0.2 mg/ml streptavidin in blocking buffer into the channel using syringe connected to the outlet tubing (as in Step B4) and incubate for 15 min. A volume of 100 μl of solution is sufficient to fill all five channels. From this step onwards it is very important to avoid letting air into the channels as exposure to air could degrade the modified surface, so always keep the inlets in a solution.
  7. Rinse the channel with HBS pH 7.5 to remove the streptavidin solution (~200 μl per channel). Use modified flow channels on the day of preparation taking care to keep the channels filled with solution.
  8. Place the microfluidic device onto the microscope stage and connect the outlet tubing of the channel to be imaged to a syringe pump. Operate the syringe pump in "withdraw" mode to pull solutions through the channels.
*Note: The flow rates can vary from the values suggested below without damaging the modified surface (typically in the range between 5 and 100 μl/min), whereby slow flow rates allow for more time for particle to diffuse and bind to the surface (e.g., in Step B9) while faster flow rates result in more efficient removal of non-specifically bound particles (e.g., in Step B11) and faster solution exchange rates.*
  9. Inject purified biotinylated viral particles in HBS pH 7.5 through the channel at a flow rate of 25 μl/min until a density of ~1,000 viral particles per field of view is obtained (typically 30-100 μl).
  10. Place the objective at the center of the channel and focus on the surface. You should see the fluorescent viral particles bound to the surface as bright diffraction-limited spots.
  11. Wash-out the unbound viral particles with 50-100 μl of HBS pH 7.5 at a flow rate of 65 μl/min.
- Perform capsid uncoating assay via painting with CypA
  1. Prepare 40 μl of CypA paint solution: HBS pH 7 containing PFO (200 nM), 0.5-1 μM of AF568-CypA and an oxygen quenching system (2 mM trolox, 2.5 mM protocatechuic acid (PCA) and 0.25 U/ml protocatechuate-3,4-dioxygenase (PCD)) to reduce photobleaching during the dual color experiments. The pH of the imaging buffer is adjusted to pH 7 because PFO is considerably less efficient at higher pH (*e.g.*, pH 7.5).
  2. Initiate the assay by injecting 30 μl of CypA paint solution at a flow rate of 65 μl/min.
  3. At the same time start acquiring TIRF images sequentially with 488 nm and 561 nm laser excitation. Example images are shown in Figure 5 and a typical image stack is shown in Videos 1 and 2.
*Notes:*
    - *
Laser power and exposure time should be chosen to obtain a signal-to-noise ratio that is sufficient to detect the level of GFP trapped inside the capsid (see below) while minimizing the energy that the sample is exposed to. High laser powers and long exposure times can lead to photochemically induced damage that affect the uncoating kinetics. On our system we typically use a power density of ~1 W cm^-2^ (measured at the objective with the laser beam normal to the surface of the coverslip) and an exposure time of 20 ms. These settings avoid saturating the CCD camera in the GFP channel and result in a single molecule photobleaching rate of 0.02 s^-1^ (half-life of 35 s) in the AF568-CypA channel.
*
    - *The frame rate and total imaging time depend on the experimental question; we typically record capsid opening traces by acquiring 800 frames, e.g., at a frame rate of 1 frame/s (total imaging time of 13.3 min) for short experiments or at a frame rate of 1 frame/6 s (total imaging time of 80 min).*
    **Figure 5.**
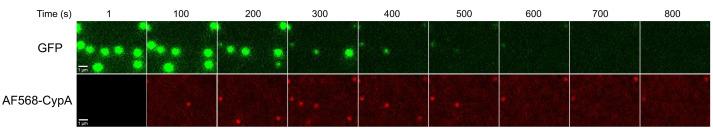
Capsid uncoating assay via painting with CypA. Snapshot images from a time series of viral particles recorded in the GFP channel (content marker, top) and the AF568 channel (CypA paint, bottom) at 1 frame per second. Snapshots correspond to indicated time points from the experiment shown in Video 2. Scale bars = 1 μm.
    **Video 1.**
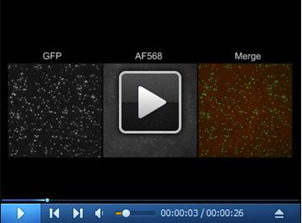
Capsid uncoating assay via painting with CypA. Image stack of the GFP channel (content marker), AF568 channel (CypA paint), and merge of both videos of a typical capsid uncoating assay via painting with CypA (complete field of view, 1,024 x 1,024 pixels). Images were acquired at 1 frame per second.
    **Video 2.**
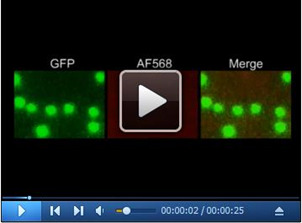
Capsid uncoating assay via painting with CypA. Image stack of the GFP channel (content marker), AF568 channel (CypA paint), and merge of both videos of a typical capsid uncoating assay via painting with CypA (80 x 60 pixel region of the field of view). Images were acquired at 1 frame per second.
  4. Repeat this procedure in the remaining flow channels of the microfluidic device.
- Single molecule photobleaching of AF568-CypA
  1. Mount a clean coverslip in a magnetic chamber and add ~300 μl of 0.02 mg/ml BSA to passivate it. Incubate for 2 min and remove.
  2. Wash with HBS 7 and add a solution of 10 nM of AF568-CypA in HBS pH 7. Incubate for 3-5 min.
  3. Remove unbound molecules by washing with HBS 7 and mount the chamber on the microscope stage for imaging.
  4. Acquire photobleaching movies in different fields of view with a 561 nm laser with the same laser power used during the uncoating assay and exposure times of 200 ms until most of the single molecules are photobleached (~150 frames).

## Data analysis

**Figure 7.**
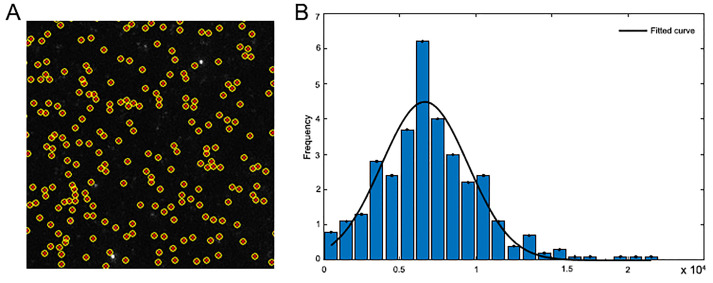
Single molecule photobleaching of AF568-CypA. A. TIRF image of AF568-CypA molecules adhered to the coverslip surface; particles detected by the image analysis software are marked with a red dot and a yellow circle. B. Representative distribution of step height from single-molecule photobleaching traces. The black line represents a Gaussian fit of the distribution.

Analysis is performed in three stages:

1. Creation of single particle traces using the Generate_Multi_Channel_Traces program in the JIM microscopy suite:
  - The two channels are aligned to each other and drift correction is calculated.
  - A subaverage of the start of the experiment is made to detect all particles.
  - Particles are detected using a threshold. Clusters of viral particles are excluded by setting the maximum eccentricity to 0.4. Background noise is excluded by setting the minimum number of pixels of a detected particle to 10.
  - The area around each particle is expanded by 4 pixels to ensure that all fluorescence is captured. A region with a radius of 20 pixels further surrounding the particle is used to calculate the background fluorescence.
  - The fluorescence intensity in each frame is calculated by taking the total fluorescence in each particle ROI minus the background fluorescence intensity.
2. Trace analysis
  - Identify the time and the height of GFP release steps (decrease in signal intensity) in each GFP trace (Böcking *et al.*, 2011). This can be automated using a step fitting algorithm (*e.g.*, in MATLAB).
  - Sort the traces into different categories depending on the number of steps the signal has, according to the following classification criteria: (1) loss of entire GFP signal in one step (“leaky” particles, Figure 6A); (2) loss of GFP intensity in one large (permeabilization) and one small (capsid opening) step (“opening” particles, Figure 6B); (3) loss of the majority of the GFP signal in one step with residual GFP signal persisting for the rest of the experiment (“closed” particles, Figure 6C); (4) no permeabilization; (5) uninterpretable traces. Sorting can be automated by defining thresholds for the different intensity levels. The assignment to classes should be verified by visual inspection of traces.
  - To plot lattice disassembly of leaky capsids, align the individual binding traces from class 1 at the time of membrane permeabilization. To plot lattice disassembly of capsids that undergo uncoating during the experiment, align traces from class 3 at the time of core opening.
  **Figure 6.**
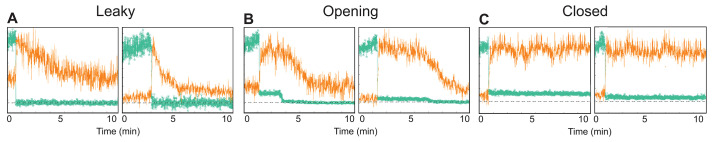
Capsid uncoating assay via painting with CypA traces. Example traces of CypA binding (vermillion) to individual leaky (A), opening (B) and closed (C) capsids that retain or do not retain GFP (green) after permeabilization of the viral envelope.
3. Single molecule photobleaching intensities are quantified using JIM.
  - Photobleaching traces are generated using the Generate_Single_Channel_Traces program.
  - Traces are stepfit to identify particles with single steps (representative of single molecules) using the Single_Molecule_Photobleaching method.
  - The step-heights of single step traces are fit to a gamma distribution and the modal intensity is taken as the single molecule intensity. A representative distribution of single step traces with fit is shown in Figure 7.
  - All traces from the CypA binding experiments are divided by this value to convert the traces from fluorescent intensity to the number of molecules.

## Recipes

1. Cell culture media
  Dulbecco’s Modified Eagle Medium (DMEM) supplemented with 10% fetal bovine serum (FBS)
2. HBS
  50 mM HEPES
  100 mM NaCl
  Adjust to pH 7.0 or 7.5
3. Lysis buffer
  25 mM HEPES, pH 7.6
  1 mM DTT
0.02% w/v NaN_3_
  1 mg/ml lysozyme
  Supplemented with "Complete" protease inhibitor
4. AIEX buffer A
  25 mM HEPES, pH 7.6
  1 mM DTT
0.02% NaN_3_
5. AIEX buffer B
  AIEX buffer A
  1.5 M NaCl
6. CIEX buffer A
  25 mM sodium phosphate pH 5.8
  1 mM DTT
0.02% NaN_3_
7. CIEX buffer B
  CIEX buffer A
  1 M NaCl
8. CypA storage buffer
  25 mM MOPS, pH 6.6
  1 mM DTT
0.02% NaN_3_
9. AF568-CypA storage buffer
  50 mM Tris, pH 8
  20% v/v glycerol
  1 mM DTT
10. Blocking buffer
  20 mM Tris pH 7.5
  2 mM EDTA
  50 mM NaCl
0.03% NaN_3_
  0.025% Tween 20
  0.2 mg/ml BSA
